# Open Letter to Dr. Fred. L. Bogue, Editor the New York Institute of Stomatology

**Published:** 1901-04

**Authors:** B. F. Arrington

**Affiliations:** Goldsboro, N. C.


### OPEN LETTER TO DR. FRED. L. BOGUE, EDITOR THE NEW YORK INSTITUTE OF STOMATOLOGY.

Dear Sir,—I find in the International Dental Journal, February issue, 1901, page 103, Report of Society Meetings, November 9, 1900, mention made by Dr. George H. Maxfield, of Holyoke, Mass., with very strong endorsement, which I am glad to see, of the practice (presumably new, but of many years' growth) of tin fillings capped with gold by Dr. T. D. Shumway, of Plymouth, Mass. I write to inform you that the same line of practice, except the use of Ivory pluggers, has been that of some dentists many years (more than thirty), and mention of the practice and recommendation of it has been made in dental journals, and there has been considerable correspondence and instruction imparted on the subject.

Dr. E. K. Wedelstaedt, of St. Paul, Minn., indulged the practice of tin base and gold capping some years ago, and doubtless can impart intelligent and profitable information of experience and results pertaining to that particular mode of practice. I recollect corresponding with him some years ago in an effort to secure knowledge of the make (form) of tin he used in such work.

Teeth to my own knowledge that were filled in that way more than twenty years ago are now doing good service.

There may be special virtue in Ivory pluggers, and, with the use of them in manipulating the combination, results may be secured that cannot be with steel pluggers and burnishers, but I cannot see it, and I question if any one else can.

A cavity filled two-thirds, three-fourths, or four-fifths with tin and capped with gold is good practice. The operation of filling is more easily executed with less discomfort and tax upon nerves of patients, and is more tooth-preserving, and is unquestionably a more conservative and progressive practice than the *old-rut, one-idea practice* of all gold from base to finish or nothing.

My experience of many years in the practice of tin base and gold capping enables me to speak favorably of the combination. Results entirely satisfactory justify the assumption that it is a line of practice in which practical instruction should be liberally dispensed in dental colleges.

B. F. Arrington.

Goldsboro, N. C.

